# Enzymatically Active Polydopamine @ Alkaline Phosphatase Nanoparticles Produced by NaIO_4_ Oxidation of Dopamine

**DOI:** 10.3390/biomimetics3040036

**Published:** 2018-11-12

**Authors:** Salima El Yakhlifi, Dris Ihiawakrim, Ovidiu Ersen, Vincent Ball

**Affiliations:** 1 Institut National de la Santé et de la Recherche Médicale, Unité Mixte de Recherche 1121, 11 rue Humann, CEDEX, 67085 Strasbourg, France; salima.elyak@gmail.com; 2 Institut de Physique et de Chimie des Matériaux, UMR 7504 CNRS—Université de Strasbourg, 23 rue du Loess, BP 43, CEDEX 2, 67034 Strasbourg, France; dris.ihiawakrim@ipcms.unistra.fr (D.I.); ovidiu.ersen@ipcms.unistra.fr (O.E.); 3 Faculté de Chirurgie Dentaire, Université de Strasbourg, 8 rue Sainte Elisabeth, 67000 Strasbourg, France

**Keywords:** melanin-like nanoparticles, sodium periodate, enzymatic activity, layer-by-layer films

## Abstract

Polydopamine (PDA) deposition, obtained from the oxidation of dopamine and other catecholamines, is a universal way to coat all known materials with a conformal coating which can subsequently be functionalized at will. The structural analogies between polydopamine and eumelanin, the black-brown pigment of the skin, were incited to produce stable polydopamine nanoparticles in solution, instead of amorphous precipitates obtained from the oxidation of dopamine. Herein, we demonstrate that size-controlled and colloidally stable PDA-based nanoparticles can be obtained in acidic conditions, where spontaneous auto-oxidation of dopamine is suppressed, using sodium periodate as the oxidant and a protein, like alkaline phosphatase (ALP), as a templating agent. The size of the PDA@ALP nanoparticles depends on the dopamine/enzyme ratio and the obtained particles display enzymatic activity of alkaline phosphatase, with an activity extending up to two weeks after particle synthesis. The PDA@ALP nanoparticles can be engineered in polyelectrolyte multilayered films to potentially design model biosensors.

## 1. Introduction

Inspired by the adhesion of mussels [1] to the surface of wood, or stones in a wet environment, dopamine [2] and other catecholamines [3,4] were proven to be interesting molecules to coat the surface of all known materials, with a conformal coating having an adjustable thickness from a few nm to more than 100 nm. The oxidation of dopamine and its subsequent self-assembly/polymerization [5,6] is at the origin of the deposition of those “polydopamine” (PDA) films on various substrates. In the presence of Tris buffer at pH 8.5, the film thickness saturates at 40–45 nm after about 16 h of auto-oxidation in the presence of dopamine at 2 mg∙mL^−1^ [2]. Thicker, but also rougher, films can be obtained by further increasing the dopamine concentration in otherwise identical conditions [7]. A major drawback of PDA and related films is the slow deposition kinetics. This problem was addressed and partially solved by depositing PDA by means of spray deposition [8], or using stronger oxidants than the oxygen dissolved in water [9,10,11].

The resulting PDA films can be deposited at all interfaces, even at water–air interfaces [12], to get flexible and asymmetric membranes in the presence of polyamines [13], or catechol-bearing polymers [14] in the aqueous subphase. In all cases, however, the deposition of conformal PDA films is accompanied by the precipitation of an amorphous and useless precipitate.

Inspired by the structural analogies between PDA and eumelanins [15,16], the black-brown pigment of the skin and dark hairs, and the homogeneous hierarchical size distribution of the eumelanin grains of the skin or sepia ink [17], attempts were made to produce colloidally stable PDA nanoparticles in solution. On the one hand, synthesis in the presence of ethanol/water mixtures and using ammonia as a catalyst allows producing PDA of controlled sizes in the 100 nm range [18,19], with numerous applications in cancer therapy, owing to the photothermal effect afforded by the optical properties of PDA [20] and in biosensing [21]. Some polymers, like poly(vinyl alcohol) [22] and surfactant micelles [23], allow for the production of stable suspensions of PDA. Of major interest is to notice that eumelanin grains are always surrounded by a tightly bound protein layer [24], making the purification of eumelanins a difficult task. Melanosomal proteins, the organelle where eumelanin is synthesized, allow for increasing the rate of eumelanin formation and association with the obtained particles [25]. Therefore, some successful attempts were made to synthesize PDA in the presence of proteins. In particular, human serum albumin (HSA) was found to increase the rate of PDA formation, and to allow for the formation of stable, biocompatible nanoparticles [26]. Tyrosine-containing tripeptides allow also for controlling the formation of eumelanin particles in a sequence-dependent manner [27]. In the case of HSA, the size of those nanoparticles was dependent on the initial protein/dopamine molar ratio and, simultaneously, to the formation of nanoparticles, and the deposition of PDA films on the surface of the reaction beaker was progressively inhibited. Other proteins were found to play a similar role, and the presence of a solvent-accessible dyad of two amino acids, namely l-lysine and l-glutamic acid, was found to play a crucial role in the formation of PDA nanoparticles [28]. However, those nanoparticles were all produced at pH 8.5, by oxygen-triggered auto-oxidation of dopamine, a pretty slow reaction mechanism.

Herein, we are wondering if the templating role of proteins can be conserved in the presence of a strong oxidant like sodium periodate, which was shown to allow for the formation of PDA films of about 100 nm thickness in only two hours [11]. In addition, sodium periodate allows for the formation of dopaminochrome exclusively from dopamine through a two-electron oxidation process [11], a much simpler reaction mechanism than in the presence of O_2_ as the oxidant. As a model protein, we choose alkaline phosphatase which was shown to allow to produce protein-rich PDA nanoparticles at pH 8.5 in the presence of dissolved oxygen [28].

## 2. Materials and Methods

### 2.1. Chemicals and Solutions

All chemicals were purchased and used without further purification. Dopamine-hydrochloride (ref. H8502), sodium periodate (NaIO_4_, ref. 311448), anhydrous sodium acetate (ref. W302406), *p*-nitrophenyl phosphate (PNP, ref. N7653), and alkaline phosphatase from bovine intestinal mucosa (ALP, ref. P7640) were all purchased from Sigma-Aldrich (L’isle D’Abeau Chesnes, France). Tris(hydroxymethyl)aminomethane (Tris buffer) was purchased from Euromedex (Schiltigheim, France). All solutions were made from ultra-pure water (Milli Plus, Millipore, Billerica, MA, USA). Dialysis tubing, with a molecular weight cut-off at 300 kDa, was purchased from Spectrum Labs (Replingen group, Waltham, MA, USA).

Two buffers were used: 50 mM sodium acetate buffer at pH 5.0, and 50 mM Tris buffer at pH 8.5. The pH of each buffer was adjusted with concentrated hydrochloric acid, and checked with a Hannah 802 pH meter, calibrated in the pH range between 4.0 and 7.0, or 7.0 and 9.0, for the sodium acetate and Tris buffer, respectively.

The diluted PNP solutions (from 5× to 100×) were solubilized in Tris buffer. The NaIO_4_ (at 20 mM) and the dopamine solutions were freshly prepared before each experiment in sodium acetate buffer.

### 2.2. Synthesis and Characterization of Polydopamine Nanoparticles

Alkaline phosphatase was first dissolved in sodium acetate buffer without any stirring. Then, dopamine at 2 mg∙mL^−1^ (10.6 mM) was added under continuous stirring. Directly after that, a small amount of NaIO_4_ solution was added, always under continuous stirring, for 6 h at room temperature (17–20 °C). The final concentration of oxidant was 20 mM in all experiments, whereas the ALP concentration was changed from 0 (reference experiment) to 4 mg∙mL^−1^. Since dopamine does not absorb at λ = 500 nm, and this wavelength is characteristic of the maximal solar emission, we followed the oxidation kinetics of dopamine in the absence or presence of ALP at this particular wavelength using an ultraviolet-visible (UV) mc^2^ spectrophotometer (SAFAS, Monaco, Monaco), with the reference cuvette containing sodium acetate buffer.

After synthesis, the solution was put in dialysis tubing and dialyzed against Tris buffer for approximately 24 h, and with three buffer changes. At the end, the solution of PDA particles was either stored at 4 °C in a glass beaker, or immediately used for characterization experiments. The particles obtained in the presence of ALP will be referred to as PDA@ALP in the following.

The colloidal stability of the nanoparticles was investigated as a function of their storage time at 4 °C, after dialysis with a NanoZS device (Orsay, France) to measure their electrophoretic mobility. The electrophoretic mobility was subsequently converted in a zeta potential value using the Smoluchowski approximation. This approximation is justified, a posteriori, for nanoparticles with a diameter larger than 30 nm in the sodium acetate buffer having an ionic strength of 32 mM at pH 5.0.

For cryo-transmission electron microscopy (TEM) characterization, a drop of the dialyzed PDA@ALP solution was deposited onto an electron microscopy grid covered by a hydrophobic carbon membrane. The drop size was progressively reduced, in order to obtain a thin film covering the whole membrane. The grid was subsequently plunged into ethane at liquid nitrogen temperature. By maintaining the specimen at this temperature, the grid was transferred on the cryo-holder, and inserted in the column of the electron microscope. These grids were analyzed by scanning transmission electron microcopy (STEM) (JEOL 2100F, Akishima, Japan) working at 200 kV, and equipped with a probe aberration corrector, an electron energy loss (EELS) spectrometer (Gatan Tridiem, Pleasanton, CA, USA), and an energy dispersive X-ray (EDX) spectrometer based on Si(Li) detector. This set-up allows reaching resolutions of 2 and 1.1 Å under TEM and STEM modes, respectively. For limiting the irradiation damage, the images were acquired using a low-density electron beam.

### 2.3. Enzymatic Activity of the PDA@ALP Nanoparticles

The enzymatic activity of the solutions containing the PDA@ALP nanoparticles was measured by following the production of *p*-nitrophenol from the hydrolysis of *p*-nitrophenol phosphate. The mother solution of PNP had a concentration of (4.6 ± 0.4) × 10^−4^ mol∙L^−1^, as determined using a method described elsewhere [29]. To determine the activity of the particles, the absorbance of solutions containing PNP and PDA@ALP mixtures was measured with a double-beam UV–vis mc² spectrophotometer (SAFAS) at a wavelength of 405 nm, taking an absorbance measurement every 10 s. The reference cuvette contained a mixture of PNP (3 mL) and Tris buffer (1 mL) to account for the spontaneous hydrolysis of PNP. The studied solution was prepared with 3 mL of PNP and 1 mL of nanoparticles (1 mL of buffer in the reference cuvette). The influence of both the substrate concentration and the dilution of the nanoparticles were investigated. The kinetic experiments covered a period of 15 min. For investigating the evolution of the enzymatic activity with storage time, the suspension of nanoparticles was stored at 4 °C between two successive measurements. As a control experiment, the activity of ALP in the presence of NaIO_4_, a strong oxidant able to oxidize amino acids close to the active center of the enzyme, was investigated. An exponential decrease law was fitted to the experimental kinetics as expected for a reaction of pseudo first-order, with respect to the substrate:

(1) $$A_{405\;nm}\left(t\right)=A_{max}\times \left(1-e^{-kt}\right),$$

where *A_max_* and *k* are the maximal measured absorbance (corresponding to consumption of all the substrate) and the rate constant, respectively.

### 2.4. Layer-by-Layer Deposition of the PDA@ALP Nanoparticles and Enzymatic Activity in the Immobilized State

The negatively charged (as inferred from zeta potential measurements) PDA@ALP nanoparticles, obtained in the presence of ALP at 1 mg∙mL^−1^ after 6 h of dopamine oxidation and intensive dialysis against Tris buffer, were used to deposit polyelectrolyte multilayer films [30,31] in alternance with poly(allylamine) at 1 mg∙mL^−1^ in the same buffer as a polycation. The films were deposited on plasma-cleaned quartz slides (4 cm × 1 cm) (Thuet, Blodelsheim, France) using a deposition time of 5 min per deposition step, and subsequent rinsing with 50 mM Tris buffer. The absorption spectrum of the dried films was measured with an UV–vis mc^2^ spectrophotometer every two layer pairs, using a cleaned quartz slide as the reference, to follow the regular deposition of PDA@ALP nanoparticles and of poly(allylamine hydrochloride) (PAH). The film deposition always started with the adsorption of the polycation PAH, owing to the negative charge of the quartz substrate under these conditions. The morphology of the (PAH-PDA@ALP)_6_ films was investigated in the dry state by contact mode atomic force microscopy (AFM) (Nanoscope III, Bruker, Mannheim, Germany), using an MLCT-C cantilever (nominal spring constant: 0.01 N∙m^−1^). The enzymatic activity of the films was estimated as a function of the number of (PAH-PDA@ALP) layer pairs, by immersing the films in 3 mL of 20-fold diluted PNP solutions. The reference cuvette contained the same PNP solution in Tris buffer at pH 8.5, to compensate for the spontaneous hydrolysis of PNP.

## 3. Results and Discussion

The dopamine solution was initially transparent at pH 5.0, for which the oxygen triggered auto-oxidation is an extremely slow process. After addition of the oxidant, NaIO_4_, either in the absence or in the presence of ALP, the solution turned yellow instantaneously, and then red, which is characteristic of the formation of dopaminochrome [11] and, finally, black after 4 h of oxidation. We observed that the reproducibility of the particle preparation was strongly dependent on the rate of the oxidant addition. Indeed, to reach a final concentration in NaIO_4_, a concentrated solution of the oxidant was titrated in the dopamine- and enzyme-containing solution and, locally, the oxidant concentration is higher than expected at the first instants of the oxidation process, even under vigorous stirring. This may affect the whole particle formation process. For these reasons, we report the data from different particles batches with different symbols.

**Figure 1:**
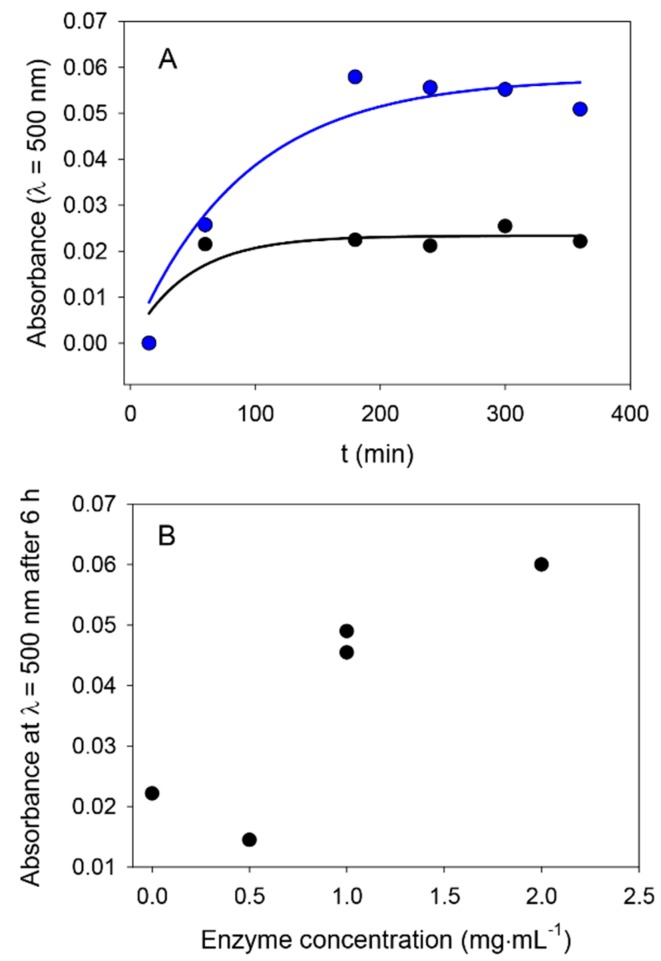
Investigation of the influence of ALP on dopamine oxidation. (**A**) Evolution of the absorbance at 500 nm at pH 5.0 in the presence of 20 mM NaIO_4_ in the absence of enzyme (⬤) and in the presence of ALP at 1 mg∙mL^−1^ (⬤). The initial dopamine concentration was equal to 2 mg∙mL^−1^ in all cases. The solid lines correspond to an exponential decay fit to the data with rate constants of (2.3 ± 0.3) × 10^−2^ min^−1^ and (5.8 ± 0.6) × 10^−2^ min^−1^ in the absence and in the presence of ALP at 1 mg∙mL^−1^, respectively. (**B**) Evolution of the absorbance after 6 h of dopamine oxidation (2 mg∙mL^−1^ at pH 5.0) as a function of the concentration of initially added ALP. Each data point corresponds to an individual curve as those described in (**A**).

From a more quantitative point of view, we investigated the kinetics of the formation of nanoparticles in the presence, and in the absence, of ALP by UV–vis spectroscopy. An illustrative experiment is shown in Figure 1, where it appears that ALP present at a concentration of 0.5 or 1 mg∙mL^−1^ is able to produce PDA aggregates faster than in the absence of the enzyme from a 2 mg∙mL^−1^ dopamine solution, compared to the reference case in the absence of enzyme. These results are similar to those obtained for dopamine in the presence of ALP, but at pH 8.5 in the absence of another added oxidant [28]. This increase in the oxidation rate of dopamine in the presence of ALP is also similar to the influence found for HSA, but at pH 8.5 in the presence of dissolved oxygen as the oxidant [26].

As an interesting finding, the absorbance reaches a saturation value after only a few hours of oxidation in the presence of NaIO_4_, whereas the process is much slower at pH 8.5 using dissolved oxygen as the oxidant [26,28]. This result is in line with the fast formation of PDA films using NaIO_4_ as the oxidant [11].

**Figure 2:**
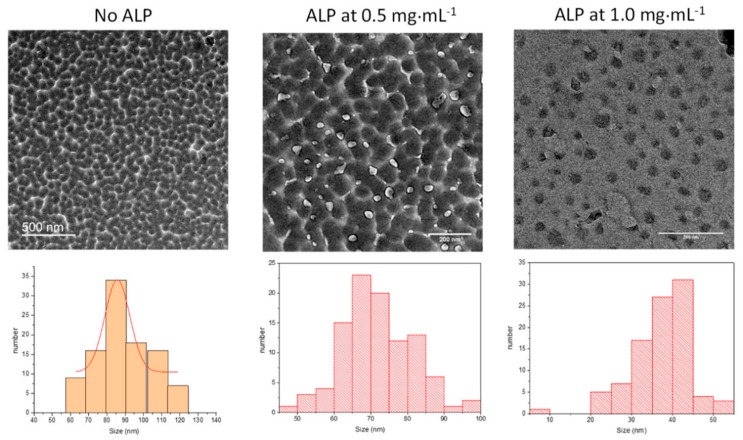
(**Top**) Representative TEM micrographs of polydopamine nanoparticles produced after 6 h of oxidation in the presence of 20 mM NaIO_4_ from the dopamine solutions (10.6 mM) after the subsequent dialysis against Tris buffer, as a function of the added concentration in ALP. (**Bottom**) Size distribution of the particles obtained by analyzing 100 particles.

The size of the nanoparticles obtained after 6 h of oxidation and subsequent dialysis of the reaction medium was obtained by TEM analysis (Figure 2). It appears, clearly, that an increase in protein concentration induces a significant reduction in the average size of the PDA nanoparticles.

In the presence of 20 mM NaIO_4_ as oxidant, the effect of the enzyme is, however, less pronounced than in the case of PDA synthesized at pH 8.5 using dissolved O_2_ as the oxidant. In this later case, the average particle size was reduced from about 500 to 50 nm when the ALP concentration was increased from 0 to 2 mg∙mL^−1^ [28]. The fact that the PDA particles are smaller when synthesized in the presence of NaIO_4_ versus O_2_ may well originate from the fact that NaIO_4_ not only allows 5,6-indolequinone to be produced in a single chemical pathway, but may also degrade the obtained polydopamine [11].

**Figure 3:**
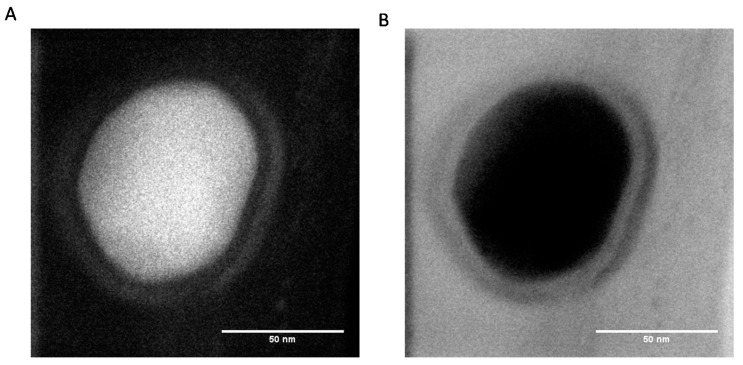
High-resolution STEM images acquired simultaneously in (**A**) high-angle annular dark field and (**B**) bright field modes of PDA@ALP nanoparticles obtained after 6 h of oxidation of a 10 mM dopamine solution + 1 mg∙mL^−1^ ALP in the presence of 20 mM NaIO_4_.

The TEM micrographs obtained at higher resolution show the presence of an external corona less dense than the core of the particles, suggesting that the composition of the external part of the particles is richer in protein than the core (Figure 3).

Nevertheless, at the present stage of our investigation, we do not have definitive proof that the obtained PDA@ALP nanoparticles are of the core–shell type. It seems that the corona of the particles may just be richer in protein than the core. However, owing the preparation method of the nanoparticles, just by mixing dopamine and the enzyme, we cannot exclude that proteins are also present in the core of the particles.

**Figure 4:**
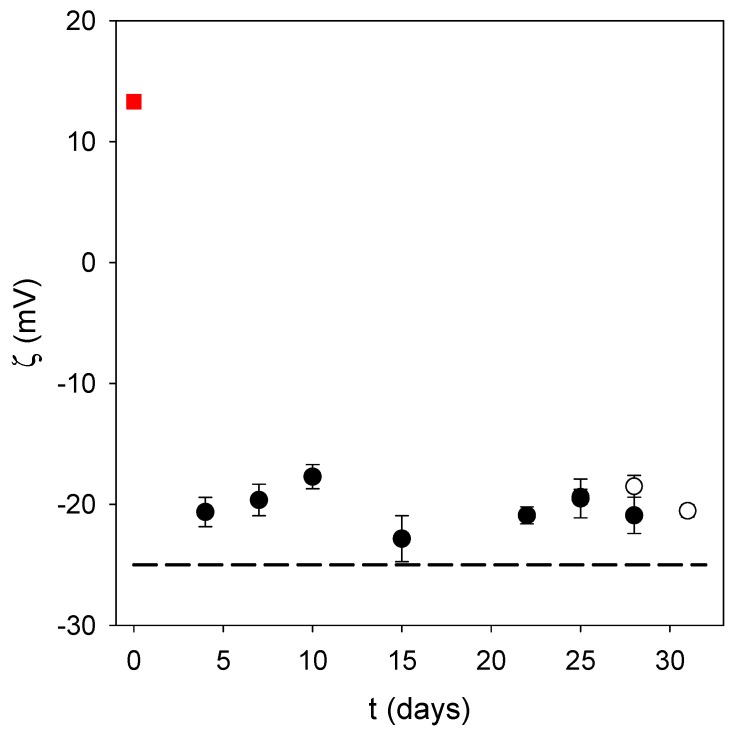
Zeta potential of PDA particles obtained after 6 h of oxidation of a 2 mg∙mL^−1^ dopamine solution in the presence of 20 mM NaIO_4_ without added enzyme (■) and in the presence of ALP at 1 mg∙mL^−1^ (two independent syntheses: ◯, ⬤). The particle suspension was dialyzed against Tris buffer as described in the Materials and Methods section. Each data point corresponds to the average over three measurements on a same batch of particles, and the error bar corresponds to one standard deviation. The dashed line corresponds to the zeta potential of a 1 mg∙mL^−1^ solution of the enzyme in Tris buffer, namely (−25 ± 5) mV.

Regardless, it appears that oxidation of dopamine in the presence of ALP in acidic conditions and using NaIO_4_ as the oxidant allows the small nanoparticles to be produced in a much faster way than at pH 8.5 under auto-oxidation conditions. This is a major advantage provided that the obtained nanoparticles are stable, and are of use for biological applications. Indeed, the obtained PDA@ALP nanoparticles were stable from a colloidal point of view, with no sedimentation during weeks, as observed visually, and a stable zeta potential during storage time (Figure 4). In addition, the zeta potential of the PDA@ALP nanoparticles was markedly different from the zeta potential of the large nanoparticles obtained in the absence of enzyme. The zeta potential of the pristine particles prepared in the presence of NaIO_4_ as the oxidant (in the presence of sodium acetate buffer at pH 5.0) is different from the zeta potential of the particles prepared using O_2_ as the oxidant directly in the presence of the Tris buffer (+(20 ± 3) mV). This difference may originate from the influence of NaIO_4_ on the composition and the structure of the obtained polydopamine. Indeed, NaIO_4_ is not only an efficient oxidant to oxidize dopamine, yielding almost exclusively dopaminochrome as a PDA precursor, but an oxidant strong enough to degrade PDA and to enrich its composition in carboxylate groups, as shown by means of infrared and X-ray photoelectron spectroscopy [11].

Even more interesting is the zeta potential reversal in the presence of ALP: pristine PDA displays a positive zeta potential at pH 5.0 in agreement with the zeta potential of PDA films produced by auto-oxidation of dopamine (but at pH 8.5), whereas PDA@ALP displays a negative zeta potential around −20 mV at pH 5.0. This finding is in agreement with the observation that ALP is present in the corona of the obtained nanoparticles (Figure 3). Indeed, the zeta potential of an ALP solution at 1 mg∙mL^−1^, in the presence of Tris buffer, is equal to −25 ± 5 mV.

The possible presence of ALP on the surface of the PDA@ALP nanoparticles prompted us to test if ALP keeps at least part of its enzymatic activity in these pretty harsh synthesis conditions.

**Figure 5:**
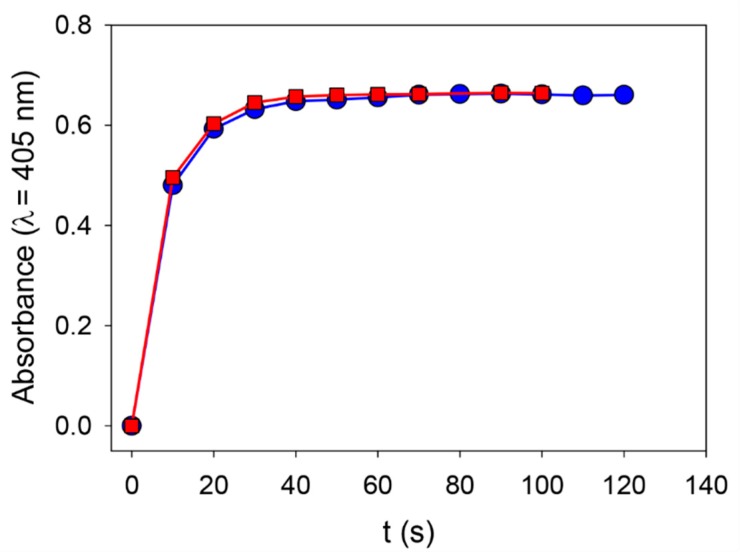
Enzymatic activity of ALP (0.1 mg∙mL^−1^) in the presence of 50 mM Tris buffer (^__^■^__^) and in the presence of 50 mM Tris buffer with 20 mM NaIO_4_ (^__^⬤^__^).

We first verified that ALP solubilized in 50 mM Tris buffer keeps its enzymatic activity when put into contact with 20 mM NaIO_4_ (Figure 5).

These findings strongly suggest that the strong oxidant does not affect the enzymatic activity of the enzyme itself, and this is reflected by the fact that the PDA@ALP particles keep the enzymatic activity expected for ALP after oxidative synthesis in the presence of NaIO_4_ and subsequent dialysis against Tris buffer at pH 8.5 (i.e., the optimal pH for ALP). All the particles prepared in the presence of ALP at different concentrations display ALP enzymatic activity; more so, the higher the concentration of enzyme, the higher the enzymatic activity. This may simply reflect an increase in the area/volume ratio of the smaller particles obtained when the oxidation of dopamine is performed in the presence of a higher concentration in enzyme. In the following we will, however, focus exclusively on the particles prepared in the presence of ALP at 1 mg∙mL^−1^.

**Figure 6:**
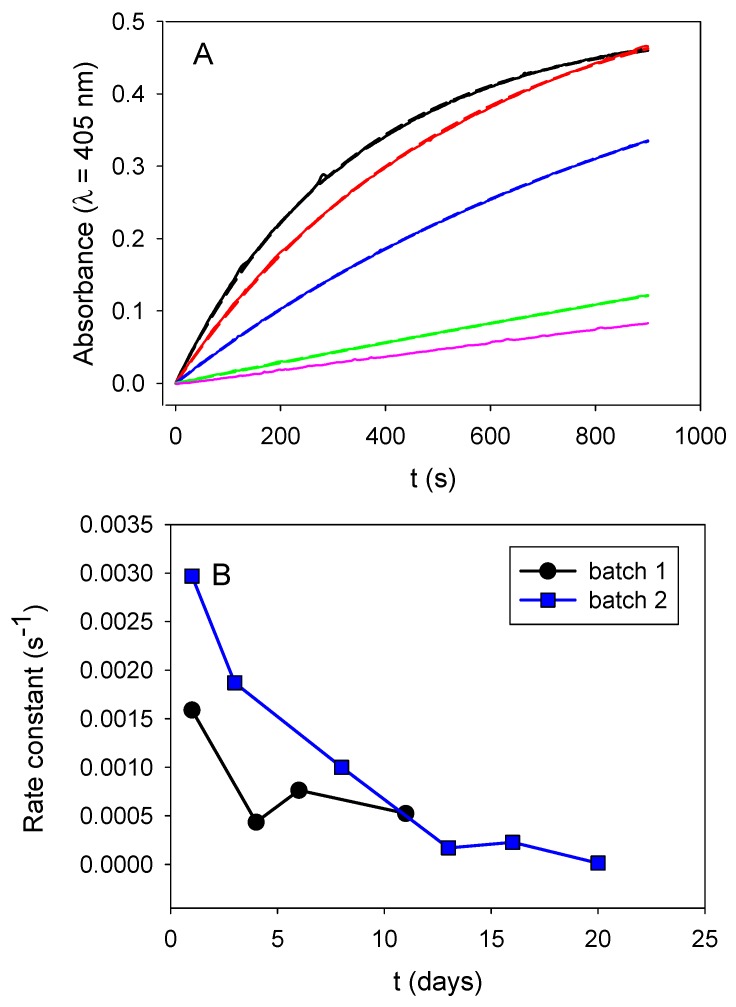
Influence of the substrate concentration and storage time on the enzymatic activity of a 10-fold diluted PDA@ALP nanoparticle suspension. (**A**) Evolution of the enzymatic activity of a 10-fold diluted PDA@ALP nanoparticle suspension in the presence of a 20-fold diluted PNP solution at day 1 (____), 3 (____), 8 (____), 14 (____), and 20 (____) after the end of the particles dialysis step against Tris buffer at pH 8.5. (**B**) Evolution of the rate constant of the enzymatic hydrolysis kinetics shown in (**A**) for a first batch of particles (corresponding to the kinetics displayed in Figure 5A (■)) and for an independent batch of particles (⬤).

Those obtained nanoparticles progressively loosed their enzymatic activity upon storage at 4 °C (between two successive measurements) (Figure 6A). This loss in activity cannot be attributed to ALP desorption from the particle’s surface, because we measured the activity of the particles in the presence of their supernatant buffer after appropriate dilution by a factor of 20. In addition, this progressive loss in activity is reproducible in at least two independent particle batches (Figure 6B). It may represent a slow and progressive enzyme denaturation when grafted on the particles’ surface.

**Figure 7:**
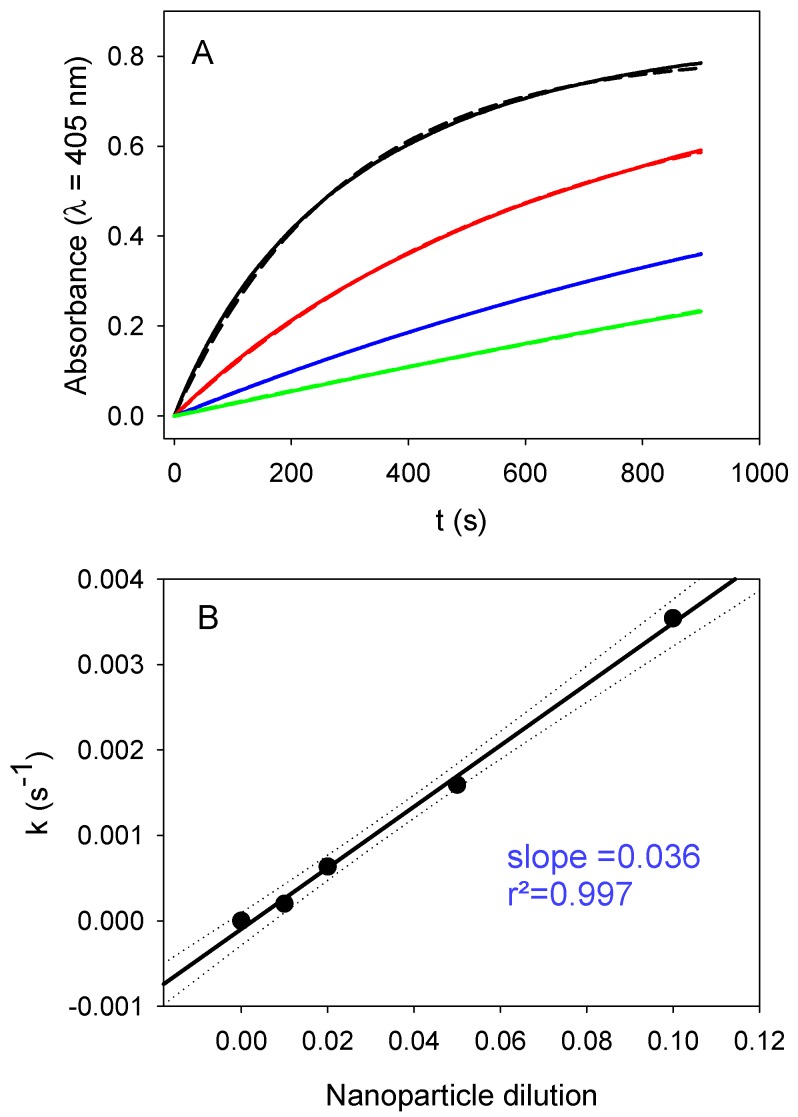
Influence of the dilution of PDA@ALP nanoparticles on their enzymatic activity. (**A**) Hydrolysis of 10-fold diluted PNP solutions in the presence of PDA@ALP nanoparticles at different dilutions: mother suspension issued from the synthesis and dialysis-diluted 10- (____), 20- (____), 50- (____), and 100-fold (____). The solid lines correspond to the experimental data, whereas the dashed lines correspond to the fit of Equation (1) to the experimental data. (**B**) Rate constant obtained by fitting Equation (1) to the experiments displayed in (**A**) versus the nanoparticle dilution factor.

Taking these results into account, we investigated the influence of both the nanoparticle relative concentration (expressed as the nanoparticle dilution in the following) and the substrate concentration on the enzymatic activity of PDA@ALP nanoparticles immediately at the end of the dialysis step against Tris buffer. The influence of the dilution in nanoparticles for a constant concentration in PNP (10-fold diluted mother solution, corresponding to an effective concentration of (4.6 ± 0.4) × 10^−4^ mol∙L^−1^), is given in Figure 7. The obtained linear relationship demonstrates that the PNP hydrolysis is a first-order process with respect to the concentration in nanoparticles and, hence, also to the enzyme concentration available on the surface of the nanoparticles, as expected. 

**Figure 8:**
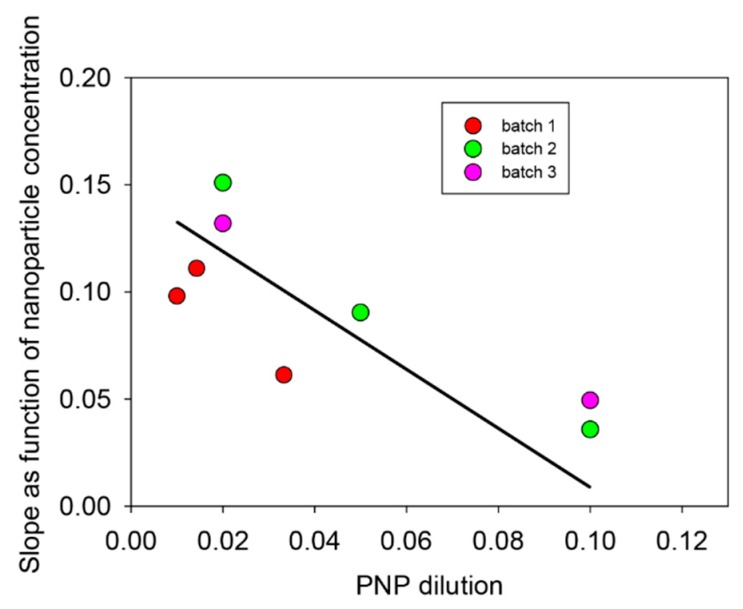
Evolution of the slope of the curves shown in Figure 7B as a function of the PNP dilution (mother solution at (4.6 ± 0.4) × 10^−4^ mol∙L^−1^) for three independent batches of PDA@ALP nanoparticles. The solid line does not correspond to a fit, but is aimed to guide the eye.

The same kinds of experiments were performed, but changing the concentration of the substrate at a given concentration of the PDA@ALP nanoparticles. The results are shown in Figure 8.

For all the investigated particle batches, the rate constant for the hydrolysis of PNP decreases with the substrate concentration, which proves that immobilized ALP on PDA nanoparticles is inhibited by its substrate.

**Figure 9:**
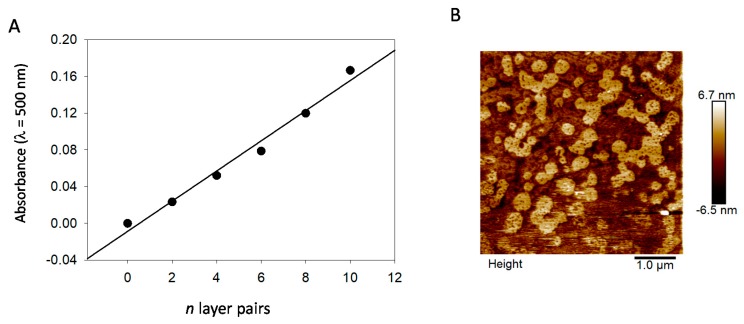
Incorporation of PDA@ALP nanoparticles in layer-by-layer films. (**A**) Evolution of the absorbance at λ = 500 nm (where PAH does not absorb light) of (PAH-PDA@ALP)*_n_* films with the number of deposited layer pairs, *n*. (**B**) Film morphology of a (PAH-PDA@ALP)_6_ film as investigated by means of contact mode AFM.

Using the finding that the PDA@ALP nanoparticles keep the enzymatic activity of the grafted enzyme, we thought to use such particles as active components in thin films which, by using other more relevant enzymes, can be useful for the design of biosensors. Layer-by-layer deposited films [30,31] offer the advantage to allow for a progressive increase in the surface concentration of active compounds, owing to a regular growth process with the number of deposition steps. The (PAH-PDA@ALP)_n_ films grow regularly with the number of deposition cycles *n* (Figure 9A), and the obtained films reflect the presence of nanoparticles close to about 100–400 nm in diameter, as inferred by AFM imaging (Figure 9B), and as expected for films made from PDA@ALP nanoparticles prepared in the presence of ALP at 1 mg∙mL^−1^. This size is in relative agreement with the particles’ size determined by TEM (Figure 2). In the immobilized state, the particles may become closer than in solution and aggregate.

**Figure 10:**
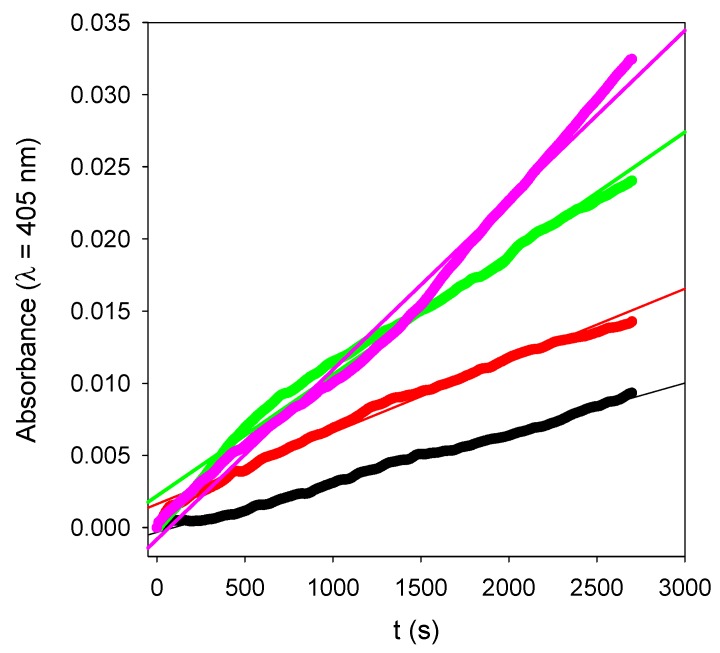
Enzymatic kinetics for (PAH-PDA@ALP)*_n_* films put in contact with a 20-fold diluted PNP solution as a function of the number of deposited layer pairs: *n* = 3 (^__^⬤^__^), *n* = 6 (^__^⬤^__^), *n* = 12 (^___^⬤^___^), and *n* = 15 (^__^⬤^__^). The data points correspond to the experimental data, whereas the solid lines correspond to linear regressions of the data.

**Figure 11:**
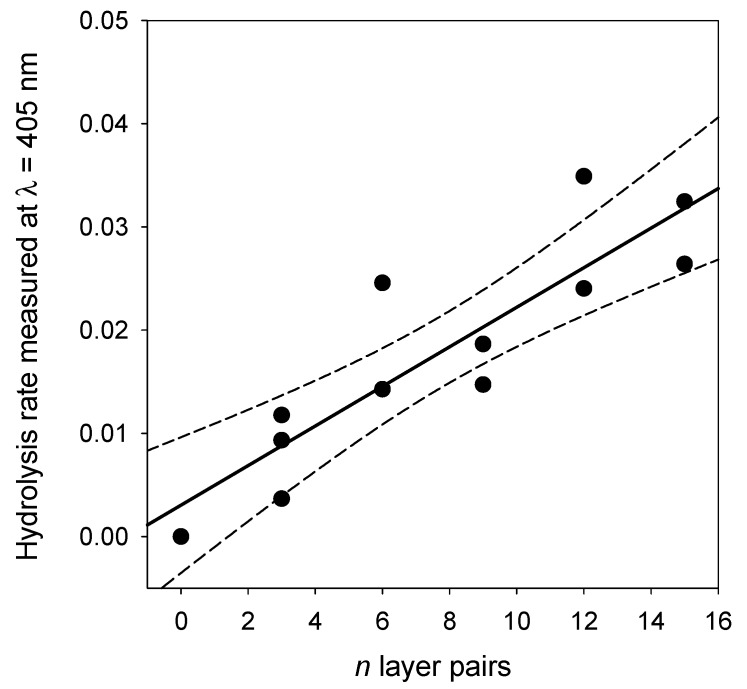
Evolution of the hydrolysis rate of (PAH-PDA@ALP)*_n_* films as a function of the number of deposited layer pairs. The solid line corresponds to a linear regression of the data points, whereas the dashed lines correspond to the limit of the 95% confidence interval.

The activity of the (PAH-PDA@ALP)*_n_* films was then measured as function of the number of deposited layer pairs, *n*, and some representative kinetics are given in Figure 10. For reaction times as short as 15 min, and for very low enzyme amounts present in the films, contrarily to the experiments performed in bulk (see Figure 7), the kinetics are linear. Such a linear regime corresponds, of course, to the first part of an exponential curve. The slope of those straight lines represents the rate of PNP hydrolysis which was then plotted as a function of the number of layer pairs in Figure 11. It appears that the hydrolysis rate of PNP scales proportionally with *n*, namely, with the film thickness (see Figure 9). This means that all enzymes on the PDA@ALP nanoparticles are accessible to the substrate. 

## 4. Conclusions

The oxidation of dopamine by NaIO_4_ in the presence of alkaline phosphatase allows for the production of stable nanoparticles of controlled size in a much shorter time than by auto-oxidation at pH 8.5. Those nanoparticles keep the enzymatic activity of the used enzyme, and seem to be enriched in enzyme on their corona with respect to their core. Owing to their negative charge, they can be immobilized in polyelectrolyte multilayer films with PAH to produce reactors with an activity proportional to the film thickness. The major challenge of this research remains to determine the accurate distribution of the enzyme in the nanoparticle to better understand the mechanism by which the enzyme controls nanoparticle formation. In addition, even if we demonstrated the possibility to produce enzymatically active PDA@ALP nanoparticles, some research effort is still required to improve the reproducibility of their preparation method. In particular, the rate of oxidant addition to the dopamine- and enzyme-containing solution should be automatized.
